# Endothelial *Zmiz1* modulates developmental angiogenesis in the retina

**DOI:** 10.3389/fcell.2025.1570587

**Published:** 2025-10-01

**Authors:** Nehal R. Patel, Shreya A. Bavishi, Adella P. Bartoletti, Rajan K C, Avery Blanks, Kelsey Carter, Mark Y. Chiang, Stryder M. Meadows

**Affiliations:** ^1^ Department of Cell and Molecular Biology, Tulane University, New Orleans, LA, United States; ^2^ Division of Hematology-Oncology, Department of Internal Medicine, Medical School, University of Michigan, Ann Arbor, MI, United States; ^3^ Tulane Brain Institute, Tulane University, New Orleans, LA, United States

**Keywords:** *Zmiz1*, transcription, co-factor, angiogenesis, retina, retinopathy

## Abstract

Angiogenesis is a highly coordinated process involving the control of various endothelial cell behaviors. Mechanisms for transcription factor involvement in the regulation of endothelial cell dynamics and angiogenesis have become better understood, however much remains unknown, especially the role of non-DNA binding transcriptional cofactors. Here, we show that Zmiz1, a transcription cofactor, is enriched in the endothelium and critical for embryonic vascular development, postnatal retinal angiogenesis, and pathological angiogenesis in a model of oxygen-induced retinopathy (OIR). In mice, endothelial cell-specific deletion of *Zmiz1* during embryogenesis led to lethality due to abnormal angiogenesis and vascular defects. Inducible endothelial cell-specific ablation of *Zmiz1* postnatally resulted in impaired retinal vascular outgrowth, decreased vascular density, and increased vessel regression. In addition, angiogenic sprouting in the superficial and deep layers of the retina was markedly reduced. Correspondingly, vascular sprouting in fibrin bead assays was significantly reduced in the absence of Zmiz1, while further *in vitro* and *in vivo* evidence also suggested deficits in EC migration. In agreement with the defective sprouting angiogenesis phenotype, gene expression analysis of isolated retinal endothelial cells revealed downregulation of tip-cell enriched genes upon inactivation of *Zmiz1*. Lastly, our study suggested that endothelial Zmiz1 is critical for intraretinal revascularization following hypoxia exposure in the OIR model. Taken together, these findings begin to define the previously unspecified role of endothelial Zmiz1 in physiological and pathological angiogenesis.

## Introduction

Vasculogenesis and angiogenesis are key processes required for the initial formation and subsequent expansion of pre-existing blood vessels, respectively. Development of a functional vasculature is critical for tissue growth, regeneration, and homeostasis, while impaired vascularization is associated with various disease processes, including stroke, vascular malformations, retinopathy, and cancer (Greenberg, 1998; Carmeliet, 2003; Crawford et al., 2009; Sherwood et al., 1971). The angiogenic generation of new blood vessels is a multi-step regulatory process intimately involving endothelial cells (ECs). For instance, vascular sprouting requires specialized ECs called tip cells that respond to both chemo-attractant and repulsive extracellular signals to guide the growth and morphogenesis of the vasculature. Functional behaviors of ECs, such as these, are regulated by transcription factors (TFs) and transcription cofactors that mediate cell-specific gene expression changes during different phases of blood vessel growth (Hamik et al., 2006; De Val and Black, 2009). However, the transcription cofactors that modulate TF involvement in the EC transcriptional program have largely remained elusive.

Zmiz1 is a transcription cofactor belonging to the protein inhibitor of activated STAT (PIAS) protein family (Liu et al., 1998). As a transcriptional cofactor, Zmiz1 does not bind DNA directly, but instead interacts with DNA-binding TFs. Zmiz1 has been shown to regulate the transcriptional activity of multiple TFs, including P53, Androgen receptor, Smad3/4 and Notch1 (Lee et al., 2007; Li et al., 2011; Li et al., 2006). Similar to the diversity of its co-partners, Zmiz1 has been shown to have roles in various developmental processes and diseases. Zmiz1 is associated with Notch-dependent T-cell development and leukemogenesis (Pinnell et al., 2015) and numerous studies point to Zmiz1 involvement in erythropoiesis (Castillo-Castellanos et al., 2021), osteosarcoma (Zhou et al., 2022), diabetes (Alghamdi et al., 2022), multiple sclerosis (Fewings et al., 2017) and in a range of neurodevelopmental disorders (Carapito et al., 2019; Lu et al., 2022; Phetthong et al., 2021). In terms of vascular development, global deletion of Zmiz1 in mice leads to lethality at embryonic day (E) 10.5 due to cardiovascular defects. Overall, the embryos are severely underdeveloped and display abnormal blood vessel development (Beliakoff et al., 2008). Further, microarray analysis of VEGF-deficient ECs displayed downregulation of *Zmiz1* in the gene ontology cluster associated with blood vessel development suggesting a functional role in the developing endothelium (Domigan et al., 2015). Related, our group recently showed that Zmiz1 transcriptionally regulates lymphatic EC gene expression (K C et al., 2024). However, the exact function of Zmiz1 in blood vessel development and angiogenesis remains unclear.

Utilizing the murine retina, a well-established model for studying the different processes of angiogenesis (Uemura et al., 2006; Stahl et al., 2010), we investigated the role of Zmiz1 in angiogenesis. In the current study, we report that loss of endothelial *Zmiz1* during embryogenesis was lethal due to vascular defects. Additionally, we demonstrated that Zmiz1 is a fundamental regulator of postnatal retinal vascular growth, including a specific and crucial function in sprouting angiogenesis. We further provided evidence of Zmiz1 involvement in the regulation of tip-cell gene expression during the vascular sprouting process. Lastly, in an oxygen-induced retinopathy (OIR) mouse model, Zmiz1 was implicated in pathological neovascularization.

## Materials and methods

### Mice and breeding

All mice were housed in individual ventilated cages, in a temperature-controlled room with a 12 h light/dark cycle. All animal protocols in this study were approved and performed in accordance with Tulane University’s Institutional Animal Care and Use Committee policies and adhere to ethical standards. In our studies the following transgenic mouse lines were used: *Zmiz1* ^fl/fl^, *Tie2*-Cre, and *Cdh5*-Cre^ERT2^ (Pinnell et al., 2015; Kisanuki et al., 2001; Wang et al., 2010). All mice were maintained in a mixed genetic background. *Zmiz1* ^fl/fl^ mice were crossed with both *Tie2*-Cre and *Cdh5*-CreERT2 lines for embryonic and postnatal studies respectively. Littermate controls of both sexes were used in all experiments. Genotyping was performed as previously detailed using genomic DNA (Huang et al., 2020).

### Tamoxifen treatment

To induce Cre activity, 100 μg of tamoxifen (Sigma, T5648) was administered orally to newborn pups born from *Zmiz1* ^fl/fl^;*Cdh5*-Cre^ERT2^ and *Zmiz1* ^fl/fl^ mating pairs from P1-P3 for early deletion or from P5-P7 for late deletion. For P42 and P60 mice, 2 mg of tamoxifen was injected intraperitoneally daily from P28-P31.

### Embryo dissection, processing, and immunofluorescent analysis

Embryos were collected from pregnant females at indicated time points following timed mating, designating embryonic day 0.5 (E0.5) as noon on the day a vaginal plug was observed. Embryos with yolk sacs were dissected and imaged by brightfield in 1X PBS, followed by overnight fixation in 4% PFA at 4 °C. Next, both yolk sacs and embryos were incubated in PBST buffer (PBS with 0.5% Triton X-100) overnight at 4 °C and then transferred into CAS-Block (Life technologies, 008120) for 4 h at room temperature. Both yolk sac tissues and whole embryos were incubated in primary antibody to rat anti-PECAM-1(BD Pharmingen, 553370) diluted in CAS-Block overnight at 4 °C. Following primary antibody incubation, both yolk sacs and embryos were incubated overnight at 4 °C with secondary antibodies to chicken anti-rat Alexa Fluor 488 (Life technologies, A21470) diluted in CAS-Block. Lastly, both yolk sacs and embryos were washed in PBST buffer before imaging. The amniotic membrane was used to collect genomic DNA for the purpose of genotyping embryos.

### Retina dissection, processing, and immunofluorescent analysis

Eyes were collected at P7, P10, P12, P42 and P60 for analysis. Retinas were dissected from eyes and whole-mount retina staining was performed as previously described (Crist et al., 2018); P60 retinas were incubated with IB4-488 for 3 h at room temperature. Primary antibodies used for whole mount retina immunofluorescent staining: IB4-488 (1:250, Invitrogen, 121411), IB4-594 (1:250, Invitrogen, 12143), SMA-Cy3 (1:250, Sigma Aldrich, C6198), ERG-488 (1:250, Abcam, Ab196374), ERG-647 (1:250, Abcam, Ab196149), COLLAGEN IV (1:100, Millipore, AB756P), NG2 (1:100, Millipore, AB5320), Ki67-488 (1:250, Cell signaling, 11882), CL-CASPASE3 (1:100, Cell signaling, 9661), ESM-1 (1:100, R&D systems, AF1999), CXCR4 (1:100, R&D systems, MAB 21651), and ZMIZ1 (1:100, Santa Cruz, Sc-376825).

### Analysis of retinal vasculature

Quantifications of retinal vasculature were performed on high-resolution confocal images using Nikon NIS-elements and Angiotool analysis software (Zudaire et al., 2011). Radial outgrowth was determined as the distance between the vascular front and the central optic nerve in each leaflet of the retina and averaged. Whole retinal images were used to measure the vascular density and the number of branching points and COLLAGEN IV sleeves (IB4 negative and COLLAGEN IV positive). Quantification of sprouts was performed at the vascular front in 20X images where white arrowheads indicate cellular protrusions identified as angiogenic sprouts.

### Isolation of murine retina and lung endothelial cells

Retinal ECs were isolated from P7 pups as previously described (Patel et al., 2022). Briefly, isolation of ECs from the retinas was performed using the Miltenyi Neural tissue Dissociation Kit (P) with minor modifications (Miltenyi, 130-092-628). For each “biological” sample, 8-10 retinas were pooled and digested in enzyme mix 1 and enzyme mix 2 to obtain a single cell suspension. Cells were incubated with CD31 conjugated dynabeads for 30 min at 4 °C. Following the incubation, CD31^+^ cells were separated from the cell mixture via magnetic activated cell sorting. Next, RNA was isolated from the cells and used for downstream RNA-sequencing. Lung ECs were isolated from P7 pups as previously described (Crist et al., 2018). Lung ECs were isolated from both control and *Zmiz1* ^iECKO^ mice and RNA was extracted for downstream assays.

### Cell culture and siRNA transfection

TeloHAECs (ATCC, CRL-4052) were cultured in EBM-2 media (Lonza, CC-3156) supplemented with EGM-2 bullet kit (Lonza, CC-3162) according to manufacturer’s instructions. All cells were maintained at 37 °C and 5% CO_2_. TeloHAECs were seeded in a 6-well plate overnight. The following day, cells were transfected with 20 μM of either pooled control-siRNAs (Dharmacon, D-001810-01-05) or *Zmiz1*-siRNAs (Dharmacon, L-007034-00-0005) using Lipofectamine3000 (Thermofisher, L3000015) following manufacturer’s instructions and were utilized for experiments 48 h post transfection. *Zmiz1*-siRNAs were purchased in the SMARTpool format, which consists of 4 different *Zmiz1*-targeted siRNAs in one reagent.

### RNA extraction and quantitative RT-PCR

RNA was extracted from TeloHAECs and isolated retina and lung ECs using the GeneJET RNA Purification Kit (Thermofisher, K0732). Nanodrop spectrophotometer measurements of RNA 260/280 absorbance ratios for RNA samples ranged from 1.90–2.09. First strand cDNA synthesis was performed using the iScript Reverse Transcription Supermix kit (Biorad, 1708840). Quantitative RT-PCR was carried out using the PerfeCTa SYBR Green Fastmix (Quantabio, 95071) and gene-specific primers (Table 1) on CFX96 system (Biorad). RT negative controls were included within the sample groups. Relative gene expression was determined using the ΔΔCt method and normalized against *β-actin*.

**TABLE 1:** qPCR Primers.

Gene (mouse)	qPCR primers
β-actin	Forward: 5′CTCTTTTCCAGCCTTCCTTC 3′
	Reverse: 5′AGGTCTTTACGGATGTCAACG 3′
Zmiz1	Forward: 5′GGAGTGACCAACACATCCC 3′
	Reverse: 5′GTTGCCACCTGGATTCATAG 3′

### Scratch assay

Scratch assays were performed on confluent control-siRNA and *Zmiz1*-siRNA treated TeloHAECs. A horizontal and vertical wound was generated in each well using a 200 μL pipet tip followed by three washes with PBS. Phase contrast images were taken right after the scratch (0 h) and 12 h after incubation at 37 °C and 5% CO_2_. The percentage of wound closure was determined using the ImageJ software.

### Fibrin gel bead assay

Fibrin bead sprouting assay was performed as previously described (Pauleikhoff et al., 2023). Briefly, control and *Zmiz1* siRNA treated teloHAECs were coated on cytodex beads and embedded into a fibrin gel. Human lung fibroblasts (NHLF) (Lonza, CC-2512) were cultured on the gels and media was replaced every other day (as previously published, fibroblasts are commonly used and necessary for efficient sprouting in the fibrin gel bead assays) (Nakatsu et al., 2007). Sprouting activity was monitored over the next few days and on day 5 the beads were imaged. Approximately 15 beads per condition were quantified for the number of sprouts per bead from 4 independent experiments. Sprout imaging and analysis were performed as described above.

### RNA sequencing and differential gene expression analysis

RNA sequencing and gene expression analysis was performed as previously described (Patel et al., 2022). Briefly, Total RNA extracted from isolated retinal ECs were quantified and verified before library preparation for sequencing. Library preparation methodology was based on mRNA polyA selection. Sequenced reads were aligned to the mouse (mm10) reference genome in the Basespace sequence hub (Illumina). The mm10 reference genome was used because our data was collected in 2019 before the release of the mm39 reference genome in 2020. The aligned reads were used to quantify mRNA expression to determine differentially expressed genes; we utilized the BaseSpace app developed by Illumina, which uses Trimmed Mean of M-values (TMM) for read counts normalization before performing differential gene expression within the pipeline. Gene ontology (GO) analysis of differentially expressed genes was performed using graphical gene-set enrichment tool for plants and animals—Shiny Go version 0.76 (Ge et al., 2020) —with a false discovery rate (FDR) set at 0.05. Sequencing data were deposited in the Gene Expression Omnibus (GEO) database: accession number GSE242406.

### OIR model

OIR studies were performed similar to previous work (Patel et al., 2022). Neonatal pups and their nursing mother were exposed to 75% oxygen from P7-P12 in a designed chamber (Biospherix, ProOx110); from P12-P17 they were moved to room air (Scott and Fruttiger, 2010). Tamoxifen (100 μg) was administered to the pups orally from P12-P14 to assess neovascularization at P17 and from P7-P9 to assess the vaso-obliteration phase at P12. Retinas were immunofluorescently labeled with Isolectin-IB4 as discussed above and vascular loss and neovascularization were quantified using an automated OIR retinal image analysis software (Xiao et al., 2017). C57BL/6J mice were subjected to either normal room oxygen (nOIR) or the OIR schedule. At P12 and P17, retinas were collected for RNA after the retinal pigment epithelium was removed. RNA isolation and quantitative RT-PCR for *Zmiz1* and *β-actin* was then performed as described above.

### Statistical analysis

Data analysis was performed using Graphpad Prism 10.3. All values are presented as bar graphs with error bars representing mean ± standard error of the mean (s.e.m). Statistical significance was determined by the two-tailed unpaired t-test between two conditions with a p-value less than or equal to 0.05 was considered statistically significant.

## Results

### Zmiz1 is expressed in the endothelium of various tissues

Given the unexplored, yet potential role of Zmiz1 in vascular development and function, we first surveyed mouse EC databases to evaluate *Zmiz1* expression in various tissues. Analysis of organ-specific EC transcriptomic data at P7 revealed highest expression of *Zmiz1* mRNA in brain ECs and comparable expression between kidney, liver, and lung ECs (Sabbagh et al., 2018) (Supplementary Figure S1A). Evaluation of single cell transcriptomic data generated using cells isolated from an adult mouse brain via fluorescence-activated cell sorting (FACS) showed a mean expression of 2.28 for *Zmiz1* in ECs^25^ (Supplementary Figure S1B). Analysis of another single-cell transcriptomic data set using isolated brain ECs from P7 mice allowed for assessment of *Zmiz1* in different EC subtypes (arterial, venous, and capillary ECs) (Sabbagh et al., 2018). *Zmiz1* expression was detected across the different EC cell types at relatively similar levels (Supplementary Figure S1C). Furthermore, bulk RNA sequencing of murine retinal ECs at different developmental stages showed the presence of *Zmiz1* transcripts during early postnatal development, with highest expression levels between postnatal days (P) 6–15, and a decrease from P21-P50 (Jeong et al., 2017) (Supplementary Figure S1D). In addition, utilizing immunofluorescence analysis, we demonstrated that ZMIZ1 protein was highly enriched throughout the blood vessels of the developing P7 retina, including the arteries, veins, and capillaries (Supplementary Figure S1E). Collectively, these studies established the presence of Zmiz1 in the endothelial lineage of postnatal and adult mouse tissues. Further, examination of human EC databases has shown *ZMIZ1* expression in both vascular and lymphatic endothelial cells (Khan et al., 2019).

### Early endothelial cell-specific ablation of *Zmiz1* results in vascular defects and embryonic lethality

**FIGURE 1:**
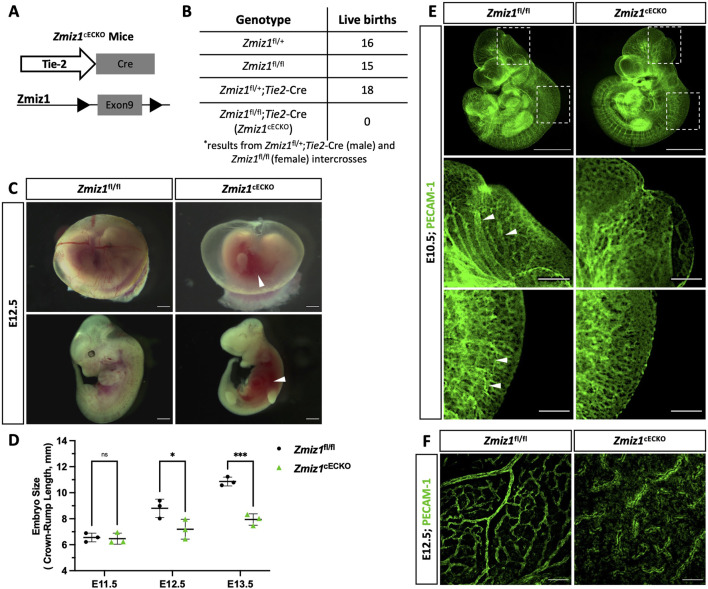
Endothelial Zmiz1 is essential for angiogenesis during embryonic development. **(A)** Strategy for *Zmiz1* exon 9 deletion specifically in the ECs during embryogenesis using the Tie2-Cre mouse model (Zmiz1 constitutive endothelial cell knockout; *Zmiz1* ^cECKO^). **(B)** Genotypic analysis of the progeny generated by crossing *Zmiz1* ^fl/+^;*Tie2*-Cre males with *Zmiz1* ^fl/fl^ females at postnatal day (P) 21. All genotypes, except *Zmiz1* ^fl/fl^;*Tie2*-Cre, were observed at normal Mendelian ratios. **(C)** Brightfield images showing gross morphology of the intact yolk sacs and whole embryos at embryonic day **(E)** 12.5. White arrowheads indicate areas of hemorrhage. Scale bars: 1 mm. **(D)** Quantification of embryo size as indicated by crown-rump length from E11.5- E13.5. **(E)** Whole-mount PECAM-1 immunofluorescent staining of E11.5 *Zmiz1* ^fl/fl^ and *Zmiz1* ^cECKO^ embryos. Scale bars 1 mm. Higher magnification of embryos shows vessel detail in the cranial and tail region (white arrowheads indicate well-organized and connected vessels in cranial and tail region). Scale bars: 100 μm. **(F)** PECAM-1 staining in E12.5 *Zmiz1* ^fl/fl^ and *Zmiz1* ^cECKO^ yolk sacs. Note the absence of distinct, organized vessels within the yolk sac of the mutants. Scale bars: 100 μm. Error bars represent mean ± s.e.m; two-tailed unpaired t-test. ns (not significant; P > 0.05), *P < 0.05, **P < 0.01, ***P < 0.001, ****P < 0.0001.

Previous work indicated that *Zmiz1* null mice die embryonically due to defects in angiogenesis (Beliakoff et al., 2008). To further investigate the endothelial role for Zmiz1 in embryonic vascular development, conditional Zmiz1 flox mice (*Zmiz1* ^fl/fl^) were crossed to mice expressing Cre recombinase under the vascular Tie2-promoter (*Tie2*-Cre) (Figure 1A), thereby achieving constitutive *Zmiz1*-EC deletion during embryogenesis. Homozygous deletion of *Zmiz1* resulted in embryonic lethality as no viable *Zmiz1* ^fl/fl^; *Tie2*-Cre (referred to as *Zmiz1* ^cECKO^; constitutive EC-knockout) pups were born (Figure 1B). Conversely, the other control genotyped pups (*Zmiz1* ^fl/+^, *Zmiz1* ^fl/fl^ and *Zmiz1* ^fl/+^;*Tie2*-Cre) were born at normal Mendelian ratios. In timed mating experiments, E12.5 *Zmiz1* ^cECKO^ embryos displayed growth retardation and were significantly reduced in size as compared to the littermate *Zmiz1* ^fl/fl^ controls (Figures 1C,D). Between E12.5 and E13.5, mutant embryos appeared pale with a lack of blood flow in the embryo, displayed an absence of blood-filled yolk sac vessels, and/or exhibited localized hemorrhages (Figure 1C). By E14.5 all the mutant embryos were necrotic and being reabsorbed (data not shown). PECAM-1 antibody staining of blood vessels in *Zmiz1* ^cECKO^ embryos at E12.5 revealed defects in the vascular patterning of the cranial and trunk vasculature (Figure 1E). In *Zmiz1* ^cECKO^ embryos, the cranial blood vessels were not well defined and failed to form larger caliber vessels, while the dorsal vasculature in the trunk, including intersomitic vessels, were truncated and disorganized. Analysis of yolk sacs at E12.5 revealed the lack of large and distinctive blood vessels in *Zmiz1* ^cECKO^ embryos in comparison to littermate controls (Figures 1C-F). Taken together, these data showed that *Zmiz1* is critical for embryonic vascular development and further indicated a major contributing role of defective angiogenesis as a cause for embryonic lethality in *Zmiz1* null mice.

### Endothelial-Zmiz1 is required for postnatal retinal angiogenesis but not vascular maintenance

**FIGURE 2:**
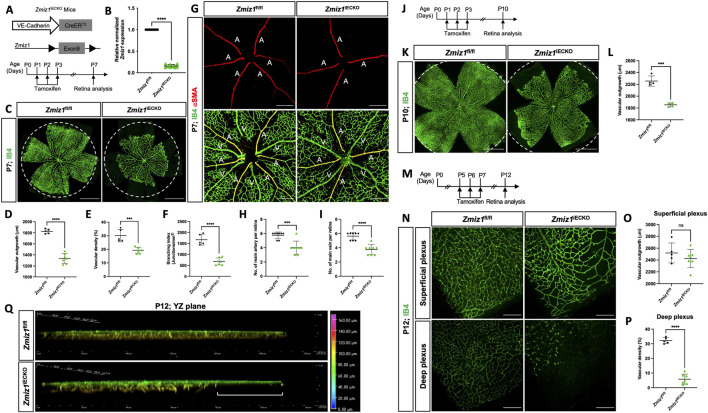
Zmiz1 EC deficiency leads to impaired retinal vascular morphogenesis. **(A)** Schematic illustration of the VE-Cadherin (*Cdh5*)-Cre^ERT2^ transgene and Cre-mediated recombination of *Zmiz1* floxed (fl) exon 9 to generate EC-specific deletion of *Zmiz1* (*Zmiz1* induced endothelial cell knockout; *Zmiz1* ^iECKO^). Time points utilized within the study for tamoxifen administration and retinal analysis are noted. **(B)** Relative *Zmiz1* expression levels in isolated lung ECs from *Zmiz1* ^fl/fl^ and *Zmiz1* ^iECKO^ mice at P7 as determined via qRT-PCR (n = 3). **(C)** Whole-mount retinas stained for isolectin-B4 (IB4). Dotted white circle represents outgrowth in *Zmiz1* ^fl/fl^ retina. Scale bar, 1,000 μm. **(D–F)** Quantification of indicated morphometric parameters analyzed within *Zmiz1* ^fl/fl^ (n = 5) and *Zmiz1* ^iECKO^ (n = 7) retinas at P7. **(G)** Whole-mount retinas stained for IB4 and alpha-SMOOTH MUSCLE ACTIN (α-SMA). Scale bar, 250 μm. A, artery; V, vein. **(H,I)** Quantification of main arteries and veins within *Zmiz1* ^fl/fl^ (n = 8) and *Zmiz1* ^iECKO^ (n = 8) retinas at P7. **(J)** Schematic showing tamoxifen administration in pups for early induction studies and analyzed at P10. **(K)** Representative images of whole-mount retinas stained with IB4 at P10. Dotted white circle represents outgrowth in *Zmiz1* ^fl/fl^ retina. Scale bars 1,000 μm. **(L)** Quantification of vascular outgrowth in *Zmiz1* ^fl/fl^ (n = 4) and *Zmiz1* ^iECKO^ (n = 4) retinas at P10. **(M)** Schematic showing tamoxifen administration in pups for late induction studies. **(N)** Representative images of IB4 vessels in superficial and deep layers at P12. Scale bars: 250 μm. **(O,P)** Quantification of vascular outgrowth and deep vascular plexus density in *Zmiz1* ^fl/fl^ (n = 5) and *Zmiz1* ^iECKO^ (n = 7) retinas at P12. **(Q)** 3D vertical view in the YZ plane with depth coding of *Zmiz1* ^fl/fl^ and *Zmiz1* ^iECKO^ retinas at P12. Note disorganized sprouts and the absence of vertical sprouting in the periphery of the *Zmiz1* ^iECKO^ retinas (white bracket). Error bars represent mean ± s.e.m; two-tailed unpaired t-test. ns (not significant; P > 0.05), *P < 0.05, **P < 0.01, ***P < 0.001, ****P < 0.0001.

To elucidate the role of Zmiz1 in the endothelium during physiological postnatal angiogenesis, we generated inducible EC-specific Zmiz1 knockout mice. This was accomplished by crossing the *Zmiz1* ^fl/fl^ line with mice carrying tamoxifen-inducible Cre recombinase under control of the EC-specific *Cdh5* promoter (Cdh5(PAC)-iCre^ERT2^) (Wang et al., 2010). Cre-mediated inactivation of *Zmiz1* in ECs (*Zmiz1* ^fl/fl^;*Cdh5*-Cre^ERT2^, referred to as *Zmiz1* ^iECKO^; *Zmiz1*-inducible EC knockout mice) was induced by daily oral administration of tamoxifen from P1-P3 for early induction (Figure 2A). Tamoxifen treatment resulted in an approximately 80% reduction of *Zmiz1* mRNA levels in isolated lung ECs (iLECs) of *Zmiz1* ^iECKO^ pups compared to littermate controls (*Zmiz1* ^fl/fl^) at P7 (Figure 2B). Retinal blood vessels were analyzed at P7 or P10 following early induction, or at P12 following late induction. Whole-mount retinas immunofluorescently labeled for the retinal endothelial cell marker, isolectinB4 (IB4), showed a significant reduction of vascular outgrowth, density, and branching in *Zmiz1* ^iECKO^ as compared to littermate controls at P7 (Figures 2C–F). Moreover, impaired retinal angiogenesis was also observed in the heterozygote mice (*Zmiz1* ^fl/wt-iECKO^), which exhibited decreased vascular outgrowth and density in comparison to the P7 controls (Supplementary Figure S2). However, the vascular defects were not as severe as those observed in the homozygous *Zmiz1* ^iECKO^ mutants. Interestingly, *Zmiz1* ^iECKO^ mice also exhibited defects in the overall number of retinal arteries and veins at P7. Immunofluorescent antibody staining of retinas with IB4 and alpha-Smooth muscle actin (α-SMA) revealed decreased numbers of main arteries and veins in *Zmiz1* mutant retinas as compared to *Zmiz1* ^fl/fl^ mice (Figures 2G–I; Supplementary Figure S2).

We also point out that though *Zmiz1* ^fl/fl^ mice treated with tamoxifen served as our controls throughout, we also assessed the retina vasculature of *Zmiz1* ^fl/fl^;*Cdh5*-Cre^ERT2^ pups that were not subjected to tamoxifen. In this case, we found that there were no differences in multiple vascular parameters assessed when comparing *Zmiz1* ^fl/fl^ and *Zmiz1* ^fl/fl^;*Cdh5*-Cre^ERT2^ mice without tamoxifen: vascular density, branchpoints and the number of major arteries and veins (Supplementary Figures S3A,C–F). However, there was a slight increase in vascular outgrowth in *Zmiz1* ^fl/fl^ retinas (Supplementary Figure S3B), while the outgrowth in *Zmiz1* ^fl/fl^;*Cdh5*-Cre^ERT2^ retinas was still similar to *Zmiz1* ^fl/fl^ mice subjected to tamoxifen (Figure 2D). Taken together, these control experiments indicated that there were no substantial vascular defects associated with potential leaking of Cre-recombinase in our studies (Supplementary Figure S3).

To further assess vascular morphology at later stages, P10 retinas from early deletion of *Zmiz1* (P1-P3) were analyzed (Figure 2J). At this time point, the vasculature in the retina has typically reached the peripheral edge. However, at P10, vascular outgrowth was strongly impaired in *Zmiz1* ^iECKO^ mice versus littermate controls (Figures 2K,L and data not shown). Thus, collectively, the early Zmiz1 deletion data demonstrated that endothelium-specific loss of Zmiz1 in postnatal mice leads to significant deficits in angiogenesis and an overall reduced vascular presence.

These initial analyses primarily focused on the retina vasculature in a two-dimensional fashion (i.e., the superficial vessels). Consequently, we evaluated the angiogenic role of Zmiz1 in three-dimensional space by analyzing deep vascular plexus formation in the retina—superficial capillaries begin sprouting vertically around P7 to give rise to the deep vascular plexus in the outer plexiform layer. Introduction of tamoxifen from P5-P7, when many of the vessels have differentiated and patterned properly but the vasculature is still remodeling and migrating to the periphery (Figure 2M), resulted in *Zmiz1* ^iECKO^ retinas with a relatively normal patterned superficial vascular plexus that reached the periphery at P12 (Figures 2N,O). However, the vascularization of the deeper vascular plexus was significantly affected, as notably fewer vessels were observed in comparison to *Zmiz1* ^fl/fl^ mice (Figures 2N–Q). Specifically, compared to control retinas, deep layer vessels were absent at the periphery in *Zmiz1* mutants, while the vessels that did invade more centrally appeared disorganized (Figures 2P,Q). Therefore, these findings indicated that *Zmiz1* is also important for the perpendicular, angiogenic sprouting events required to populate the deeper layer of the retina.

Based on the findings above, Zmiz1 is essential during periods of active retinal angiogenesis, which is consistent with its expression levels in ECs during early mouse retina development (Jeong et al., 2017) (Supplementary Figure S1D). However, the role of Zmiz1 in vascular maintenance during adulthood was unknown. To address this, we induced deletion of *Zmiz1* in adult mice from P28-P31 and analyzed retinas at P42 and P60 (Supplementary Figure S4A). Interestingly, *Zmiz1* ^iECKO^ mice did not display any obvious vascular patterning or morphological defects (Supplementary Figure S4B). Evaluation of vascular density in both the superficial and deep vascular plexi revealed no significant difference in control and *Zmiz1* ^iECKO^ mice (Supplementary Figures S4C–G). Thus, these results suggested that *Zmiz1* is not required for maintenance of vascular patterning and morphology during adulthood, which notedly corresponds to a continuous decrease in Zmiz1 endothelial cell expression from P15-P50 in the retina (Jeong et al., 2017) (Supplementary Figure S1D).

### Loss of Zmiz1 leads to increased retinal vessel regression

**FIGURE 3:**
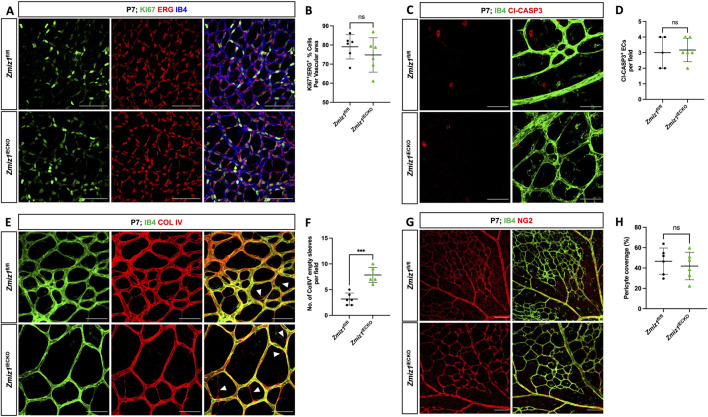
Deletion of Zmiz1 in ECs results in increased retinal vascular regression. **(A)** Representative images of P7 retinas stained with Ki67, ERG, and IB4. Scale bars: 100 μm. **(B)** Quantification of Ki67^+^ proliferating ECs (ERG+) in *Zmiz1* ^fl/fl^ (n = 6) and *Zmiz1* ^iECKO^ (n = 6) mice at P7. **(C)** Representative images of P7 retinas stained with IB4 and CLEAVED CASPASE 3 (Cl-CASP3). Scale bars: 50 μm. **(D)** Quantification of Cl-CASP3^+^ apoptotic ECs in *Zmiz1* ^fl/fl^ (n = 6) and *Zmiz1* ^iECKO^ (n = 6) mice at P7. **(E)** Representative images of P7 retinas stained with IB4 and COLLAGEN IV (COL IV; white arrowheads indicate empty COL IV sleeves devoid of ECs). Scale bars 50 μm. **(F)** Quantification of empty COL IV sleeves in *Zmiz1* ^fl/fl^ (n = 6) and *Zmiz1* ^iECKO^ (n = 6) retinas at P7. **(G)** Representative images of P7 retinas stained with IB4 and NEURAL/GLIAL ANTIGEN 2 (NG2). Scale bars 100 μm. **(H)** Quantification of pericyte coverage in *Zmiz1* ^fl/fl^ (n = 6) and *Zmiz1* ^iECKO^ (n = 6) retinas at P7. Error bars represent mean ± s.e.m; two-tailed unpaired t-test. ns (not significant; P > 0.05), ***P < 0.001.

During postnatal retinal angiogenesis, the growth of initial blood vessels to the formation of a mature vascular network involves various cellular processes, such as EC migration, proliferation, apoptosis, vessel regression, and vessel remodeling. To assess whether some of these processes were defective and contributed to the vascular phenotypes in *Zmiz1* ^iECKO^ retinas, we first analyzed EC proliferation and apoptosis. EC proliferation was assessed by co-staining for ETS-related gene (ERG; EC-specific nuclei marker) and the proliferation marker Ki67. We observed no significant difference in the rate of EC proliferation between *Zmiz1* ^iECKO^ and control retinas at P7 (Figures 3A,B). Similarly, co-staining for CLEAVED CASPASE 3 (a key protease in apoptosis) and ERG revealed that EC apoptosis at P7, which is normally low, was unaffected by loss of Zmiz1 (Figures 3C,D). Next, we assessed blood vessel regression by co-immunolabeling for IB4 and the basement matrix component COLLAGEN IV (COL IV). During vascular remodeling, COL IV sleeves devoid of ECs (COL IV positive; IB4 negative) are observed as vessels regress, and alterations in this process are typically associated with unstable, defective vascular remodeling. We identified a significant increase in the number of COL IV sleeves within *Zmiz1* ^iECKO^ retinas compared to the controls, indicative of increased vessel regression and decreased vascular stability (Figures 3E,F). However, when we assessed this at P10, we did not observe differences in vessel regression between Zmiz1 control and mutant mice (Supplementary Figures S5E,F). In addition, we examined pericyte vessel coverage since EC-pericyte interactions are also known to affect the angiogenesis process (Egginton et al., 1996; Chiaverina et al., 2019). However, immunofluorescent antibody staining for pericyte marker NEURON-GLIAL ANTIGEN 2 (NG2) and IB4 revealed no noticeable differences in pericyte coverage between the control and *Zmiz1* ^iECKO^ retinas at P7 (Figures 3G,H). Moreover, we did not identify any changes in NG2 coverage or rates of EC proliferation and death at P10 when comparing *Zmiz1* ^f/f^ and *Zmiz1* ^iECKO^ retinas (Supplementary Figure S5). These data established that EC proliferation, EC apoptosis, and pericyte coverage are unchanged in the absence of Zmiz1. Conversely, loss of *Zmiz1* in ECs resulted in substantial increases in vessel regression at P7 indicating an overall contribution of *Zmiz1* to vascular stability during angiogenic remodeling of the retinal vascular network.

### Zmiz1 mutants exhibit defective sprouting angiogenesis

**FIGURE 4:**
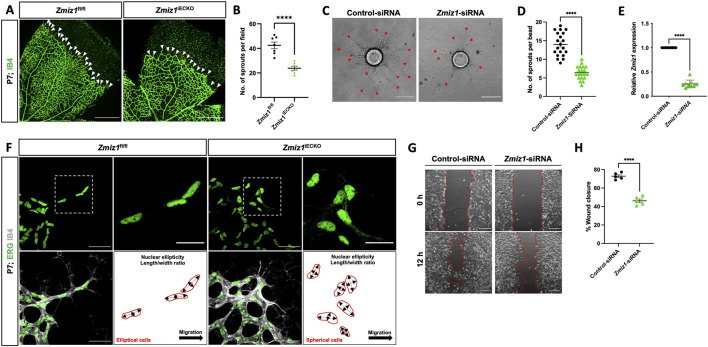
Zmiz1 is required for sprouting angiogenesis and EC migration. **(A)** Images of IB4 immunolabeled retinas at the vascular front in P7 *Zmiz1* ^fl/fl^ and *Zmiz1* ^iECKO^ mice (white arrowheads mark the leading-edge vascular sprouts). Scale bar, 250 μm. **(B)** Quantification of the number of sprouts within *Zmiz1* ^fl/fl^ (n = 7) and *Zmiz1* ^iECKO^ (n = 8) retinas at P7 (quantifications represent the average number of sprouts within each leaflet). **(C)** Representative images of bead assays embedded in 3D fibrinogen gel at 120 h following control and *Zmiz1* siRNA treatments (red arrowheads mark sprouts emanating from the bead). Scale bar, 100 μm. **(D)** Quantification of the number of sprouts per bead (n = 20). **(E)** qRT-PCR analysis of control and Zmiz1 siRNA treated TeloHAECs for *Zmiz1* mRNA levels normalized to *GAPDH* transcripts (n = 3). **(F)** Close-up images of retinas stained for IB4 and ETS related gene (ERG) to mark EC nuclei at the vascular front within P7 *Zmiz1* ^fl/fl^ (n = 7) and *Zmiz1* ^iECKO^ (n = 8) retinas. Cell-shape schematics indicating nuclear ellipticity within each group are represented. Scale bar, 50 μm and 25 μm for insets, which highlight sprouts. **(G)** Images of scratch wound assays performed on TeloHAEC confluent monolayers following control and *Zmiz1* siRNA treatments. Images at 0 and 12 h following the scratch are depicted. Scale bar, 200 μm. **(H)** Quantification of the percentage of wound closure in TeloHAECs subjected to the various siRNA treatments in G (n = 5). Error bars represent mean ± s.e.m; two-tailed unpaired t-test: ****P < 0.0001.

During initial observations of the retinal vasculature (Figure 2), we noticed an obvious difference in the sprouting angiogenic blood vessels at the vascular front between Zmiz1 control and mutant retinas. Specifically, there appeared to be fewer vessels sprouting toward the retina periphery in *Zmiz1* ^iECKO^ retinas. Indeed, quantification confirmed a significant decrease in the number of P7 vascular sprouts in Zmiz1 mutant retinas compared to controls (Figures 4A,B; Supplementary Figure S6). This reduction was notable because deficits in sprouting angiogenesis regularly result in retinal vascular phenotypes like those we observed in the *Zmiz1* ^iECKO^ retinas (Par et al., 2019; Arribas et al., 2024). Therefore, to further evaluate the role of Zmiz1 in sprouting angiogenesis, we performed the fibrin bead assay with immortalized human aortic ECs (telomerase human aortic EC; TeloHAEC). Beads were coated with equal numbers of TeloHAECs treated with either scramble-control siRNA or *Zmiz1*-targeted siRNA and allowed to sprout for 120 h. Control siRNA treated TeloHAECs showed robust sprout formation, whereas cells subjected to *Zmiz1* siRNA displayed a significant reduction in the length and number of sprouting vessels (Figures 4C,D). Quantitative polymerase chain reaction (qPCR) verified that *Zmiz1* expression was appreciably reduced in *Zmiz1* siRNA TeloHAECs (Figure 4E).

Previous reports noted defective migration of neuronal cells upon loss of Zmiz1 function *in vivo* (Carapito et al., 2019). This led us to explore the possible role of *Zmiz1* in the regulation of tip-cell behavior and migration during sprout formation/elongation—tip cells are at the leading end of the sprouting periphery vessels and direct vessel growth and migration. In this context, we first analyzed the nuclei shape of tip-cell-associated ECs at the vascular front; elliptical nuclei are often connected with directed migration, while spherical-shaped nuclei are linked to non-migratory cells (Kim et al., 2019). In control mice, ERG-positive nuclei of tip ECs were predominately elliptical in shape and directed toward the avascular area where the retinal vessels typically migrate. (Figure 4F). However, in *Zmiz1* ^iECKO^ mice, the nuclei of ECs at the leading edge were more spherical, including those in the comparatively shorter sprouts, suggesting an impaired cell migration phenotype (Figure 4F). To obtain a better grasp on Zmiz1 function in EC migration, cell migration assays (scratch assay) were carried out with TeloHAECs. Under normal culture conditions, *Zmiz1* siRNA TeloHAECs showed a decreased repopulation of cells into the “wound” area in comparison to control siRNA-treated ECs (Figures 4G,H) further suggesting defective migratory properties in the absence of Zmiz1.

### Zmiz1 inactivation leads to gene expression changes in the endothelium, including reduced expression of tip-cell-associated genes

**FIGURE 5:**
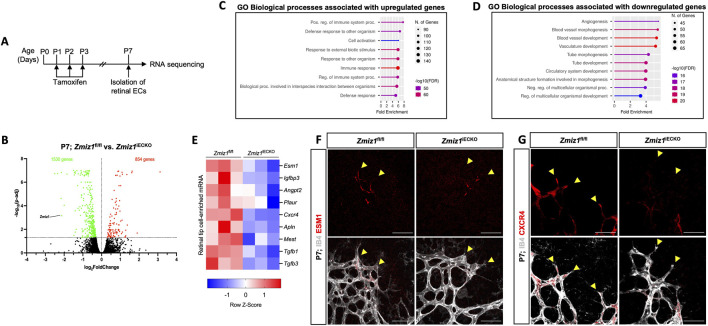
Expression of retinal endothelial tip-cell markers is downregulated upon Zmiz1 genetic ablation. **(A)** Illustration depicting the strategy used for RNA-seq analysis on isolated retinal ECs (iRECs). **(B)** MA plot of differentially expressed genes between *Zmiz1* ^fl/fl^ and *Zmiz1* ^iECKO^ iRECs at P7. Statistically significant upregulated and downregulated genes are depicted as red and green dots, respectively. Zmiz1 is highlighted and confirms reduced expression in *Zmiz1* ^iECKO^ iRECs. **(C,D)** Gene ontology (GO) analysis of biological process terms enriched in upregulated **(C)** and downregulated **(D)** genes of *Zmiz1* ^iECKO^ iRECs (false discovery rate (FDR) are shown). **(E)** Clustered heat map of gene count Z scores for retinal tip-cell enriched mRNAs. Columns represent pools of different biological samples (8-10 retinas/pool). We note that in our analyses, the floxed conditions are normalized to the iECKO conditions. **(F,G)** Close-up views of *Zmiz1* ^fl/fl^ and *Zmiz1* ^iECKO^ retinal tip-cell regions immunofluorescently labeled for IB4 and the tip-cell enriched markers ESM1 **(F)** and CXCR4 **(G)**. Scale bars, 25 μm. Notice overall reduced and sometimes absent expression of ESM1 and CXCR4 in the tip-cells (yellow arrowheads) of Zmiz1 retinas.

To further define the role of Zmiz1 in the regulation of EC behavior, we performed transcriptional profiling of isolated retinal ECs (iRECs) at P7 (Figure 5A). RNA-Sequencing (RNA-seq) analysis of three biological replicates of pooled iRECs from control and *Zmiz1* ^iECKO^ mice revealed 854 upregulated and 1,530 downregulated genes following the loss of *Zmiz1* (Figure 5B). Gene ontology (GO) analysis unveiled upregulated genes enriched for biological processes such as immune response and T-cell activation (Figure 5C), which is relevant to previous work demonstrating an important role for Zmiz1 in T cell development and leukemogenesis (Pinnell et al., 2015). Importantly, downregulated genes, representing targets that are likely positively regulated by Zmiz1, were enriched for GO terms associated with developmental processes such as angiogenesis, blood vessel morphogenesis and vascular development (Figure 5D). These transcriptional findings were in alignment with our phenotypic embryonic and retinal studies (Figures 1–4). Given our overall findings connecting Zmiz1 function to pro-angiogenic growth, especially at the retinal vascular front, we examined the iREC RNA-seq data for indications that Zmiz1 affects transcriptional regulation of tip-cell-associated genes involved in retinal angiogenesis. Analysis revealed that several notably well-characterized genes either enriched in tip-cell expression and/or involved in tip-cell formation and function, such as *Apln* (Del Toro et al., 2010), *Angpt2* (Gale et al., 2002), *Esm1* (Rocha et al., 2014), and *Cxcr4* (Strasser et al., 2010) were markedly downregulated in Zmiz1 mutant retinas (Figure 5E). Furthermore, immunofluorescent antibody stains for ESM1 and CXCR4 confirmed that expression of these tip-cell markers was notably reduced at the vascular front in Zmiz1 deficient retinas, while controls showed typical, strong expression in ECs at the leading edge (Figures 5F,G). Together, the iREC RNA-seq data and expression analysis provide further evidence that Zmiz1 is a critical tip-cell regulator required for proper retinal sprouting angiogenesis.

### Loss of Zmiz1 in an OIR retinopathy model leads to reduced neovascularization

**FIGURE 6:**
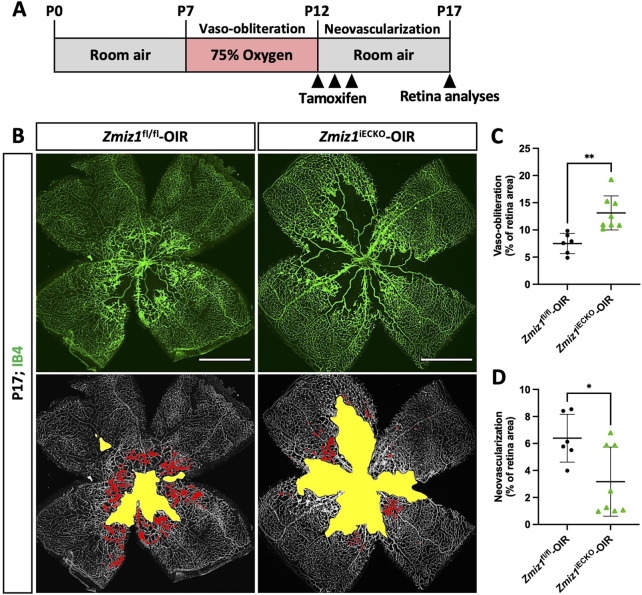
Loss of endothelial Zmiz1 impairs pathological angiogenesis. **(A)** Schematic summary of the OIR strategy implemented to assess Zmiz1 in pathological neovascularization. **(B)** Representative whole-mount images of the vasculature immunolabeled with IB4 in *Zmiz1* ^fl/fl^-OIR (n = 6) and *Zmiz1* ^iECKO^-OIR (n = 8) P17 retinas. Yellow space demarcates the avascular area, while the red markings highlight the neovascular tufts within each retina. Scale bars: 1,000 μm. **(C,D)** Quantification of the areas of vaso-obliteration (avascular space) and retinal neovascularization in *Zmiz1* ^fl/fl^-OIR (n = 6) and *Zmiz1* ^iECKO^-OIR (n = 8) P17 mice. Error bars represent mean ± s.e.m; two-tailed unpaired t-test. ns (not significant; P > 0.05), *P < 0.05, **P < 0.01.

Zmiz1 has been linked to various pathological features and diseases, such as leukemia (Pinnell et al., 2015), osteosarcoma (Zhou et al., 2022), diabetes (Alghamdi et al., 2022), erythropoiesis (Castillo-Castellanos et al., 2021), and several neurodevelopmental disorders (Carapito et al., 2019; Lu et al., 2022; Phetthong et al., 2021). To investigate whether Zmiz1 might have a role in pathological angiogenesis, we performed oxygen-induced retinopathy (OIR) studies, which are designed to emulate conditions similar to ocular retinopathies. Both *Zmiz1* control and mutant pups were housed in a hyperoxia chamber (75% oxygen) from P7 to P12 to allow blood vessel regression and death. At P12 they were transferred to normal, room oxygen levels to create a hypoxic environment that promotes neovascularization (Figure 6A). Pups were induced with tamoxifen for 3 consecutive days once placed in a normal oxygen environment (P12-14) and retinas were harvested and analyzed at P17. Compared to the *Zmiz1* ^fl/fl^ mice, Zmiz1 deficient retinas exhibited significantly larger areas of vaso-obliteration (areas devoid of vasculature) at P17 suggesting that neoangiogenesis was defective (Figures 6B–D). Accordingly, *Zmiz1* ^iECKO^ retinas displayed significantly fewer regions with neovascular tufts—areas of active growth and revascularization that occur in the OIR retinal model as the blood vessel network undergoes a corrective remodeling phase—than *Zmiz1* ^fl/fl^ retinas (Figures 6B–D). Additionally, we surveyed a previous RNA-seq data set associated with the OIR model to ascertain if *Zmiz1* expression changes during the neovascularization stage (Pauleikhoff et al., 2023). Quantification of *Zmiz1* mRNA showed that there is no statistical difference in expression between OIR and non-OIR treated C57BL6 mice at P14 or P17 (Supplementary Figure S7A–C) indicating that Zmiz1 expression is not upregulated in response to hypoxia and the generation of new blood vessels. To validate this finding, we carried out similar experiments and confirmed by qPCR that the mRNA levels of *Zmiz1* are not statistically different between OIR and non-OIR retinas in C57BL6 mice at P17 (Supplementary Figure S7D). Further, there were no significant differences in Zmiz1 expression between OIR and non-OIR subjected retinas at P12 when the vaso-obliteration process is complete (Supplementary Figure S7E).

Lastly, to assess whether Zmiz1 might have a role during the vaso-obliteration phase (P7-12), we induced deletion of Zmiz1 from P7-P9 and examined the avascular areas at P12. Interestingly, we did not observe any differences in the overall sizes of the avascular zones between *Zmiz1* ^fl/fl^ and *Zmiz1* ^iECKO^ retinas (Supplementary Figure S8). Taken together, these experimental results demonstrated that Zmiz1 is required for hypoxia-regulated angiogenesis during the revascularization process in a retinopathy setting but is not important during the vaso-obliteration step.

## Discussion

The process of new blood vessel formation is vital for organismal growth and survival. Our study highlighted an important role of Zmiz1 during embryonic and postnatal retinal vascular development. We showed that constitutive loss of endothelial *Zmiz1* during embryogenesis resulted in vascular defects and embryonic lethality. Using inducible EC-specific Cre mice, we demonstrated that angiogenesis was disrupted in *Zmiz1*-deficient ECs both in physiological and pathological settings, providing evidence of Zmiz1 as an important regulator of the developing vasculature. Specifically, early postnatal deletion of endothelial *Zmiz1* resulted in delayed retinal outgrowth of superficial vascular plexus both at P7 and P10. In addition, late induction of endothelial *Zmiz1* deletion severely impaired perpendicular vascular sprouting resulting in reduced densities of vascular plexus in the deep layer at P12. The disruption in vascular outgrowth and morphology in *Zmiz1* mutants is potentially attributed to the reduction in tip cell numbers, subsequent decreased sprout formation and reduced expression of crucial tip-cell genes. We also observed that *Zmiz1* is critical for intraretinal vascularization following ischemic injury in the OIR retina. Taken together, our studies begin to provide important clues that highlight the complex role of Zmiz1 in angiogenic development.

Although our work provides new information detailing the role of Zmiz1 in angiogenesis, a full appreciation of the transcriptional mechanisms by which *Zmiz1* regulates blood vessel growth via angiogenesis will require additional investigation. However, transcriptional profiling of *Zmiz1*-deficient ECs in this study did uncover some noteworthy targets, including *Apln* (Del Toro et al., 2010), *Angpt2* (Gale et al., 2002), *Esm1* (Rocha et al., 2014) and *Cxcr4* (Strasser et al., 2010), that are potentially and directly regulated by Zmiz1 (Figure 5). These genes are well-established to be associated with the acquisition of specialized tip/stalk-cell phenotypes in ECs during the sprouting process (Guo et al., 2024). Consequently, it is reasonable to infer that downregulation of their expression would lead to abnormal vascular development, such as that observed in our Zmiz1 mutant retinas. Future experiments focused on elucidating the precise actions of Zmiz1 on this subset of genes could offer valuable insights into the regulatory mechanisms that govern sprouting angiogenesis.

In a similar context, several prominent pathways, including VEGF-A and NOTCH signaling, are critical players regulating tip-cell and stalk-cell selection, whose ratio is critical during the angiogenic sprouting process in physiological and pathological conditions (Blanco and Gerhardt, 2013; Ubezio et al., 2016). It is plausible, for instance, that the defective patterning of arteries observed in *Zmiz1* ^iECKO^ mice is due to misregulation of the DLL4-NOTCH1 pathway as this signaling cascade is important for arteriogenesis during vascular development (Liu et al., 2003). In support of this possibility, previous work demonstrated a transcriptional partnership between Zmiz1 and NOTCH1 in the regulation of T-cell phenotypic contributions to Leukemia (Pinnell et al., 2015). Additionally, microarray analysis following inactivation of VEGF-A in cultured human umbilical vein ECs (HUVECs) revealed downregulation of Zmiz1 in the blood-vessel gene cluster (Domigan et al., 2015), which could also have an impact on tip/stalk-cell mediated growth of the vasculature. Therefore, several studies indicate a potential link between VEGF-A, NOTCH1 and Zmiz1 in regulation of sprouting angiogenesis. However, Zmiz1 may influence angiogenesis through mechanisms independent of the VEGFA and NOTCH signaling pathways, further underscoring the existing gap in our understanding of its transcriptional contributions to angiogenesis.

Our characterization of endothelial deficient mice demonstrated that the loss of Zmiz1 in ECs influenced early vascular development, as evidenced by the aberrant embryonic vasculature, and inhibited vascular expansion and impaired perpendicular branching of retinal vasculature. However, the inactivation of Zmiz1 in mature vasculature with quiescent ECs had no obvious effect on overall blood vessel integrity and remodeling. These findings suggest a more defined role of Zmiz1 in early embryonic and postnatal life, which will be essential to unraveling its molecular regulatory mechanisms. Interestingly, gene set enrichment analysis (GSEA) of gene sets identified during different retinal developmental stages revealed higher enrichment of genes associated with NOTCH signaling pathway at P6 when sprouting angiogenesis is highly prevalent in the superficial vasculature (Jeong et al., 2017). Thus, considering the connection of Zmiz1 to NOTCH signaling and the similar developmental timing, we speculate that Zmiz1 interacts with key putative TFs regulating EC behavior during the early phase of vascular development.

This study also revealed that Zmiz1 may have an important role during pathophysiological angiogenesis, as loss of Zmiz1 resulted in the reduced ability to revascularize the retina in the OIR experiments. This finding may be relevant given the building evidence linking alterations in Zmiz1 to various disease settings. For example, several patients with a Zmiz1 variant-associated syndromic neurodevelopment disorder also displayed congenital cardiovascular malformations. Although these abnormalities were not described in detail, it is tempting to postulate that they could be related to defects in the endothelial lineage. Thus, future assessment and specifics of these cardiovascular issues will be especially interesting, as our preliminary data suggest that defective Zmiz1 function in the endothelium could have a causal role in the cardiac phenotypes of these patients.

In the field of vascular biology, many TFs that are important for EC differentiation and maturation have been identified, however their mechanisms of regulation are not fully defined. This study introduces a transcription cofactor with a previously unknown specific role in vascular development and function that may be involved in regulating the activity of vascular transcription factors, including those described above. Thus, our study begins to delineate an important role of endothelial *Zmiz1* in physiological and pathological angiogenesis.

## Data Availability

The datasets presented in this study can be found in online repositories. The names of the repository/repositories and accession number(s) can be found below: https://www.ncbi.nlm.nih.gov/geo/, GSE242406.

## Supplementary material

SUPPLEMENTARY FIGURE S1 Summary of EC-specific *Zmiz1* expression within various tissues. **(A)** Analysis of *Zmiz1* expression levels in ECs and total tissue of P7 brain, kidney, liver, lung, and adult brain using the Vascular Endothelial Cell Trans-omics Resource Database (VECTRDB). **(B)** *Zmiz1* expression in each cell type isolated from an adult mouse brain can be visualized within the violin plots. **(C)** Analysis of *Zmiz1* expression levels in P7 isolated brain ECs within subtypes using single cell RNA-seq data in VECTRDB. **(D)** Expression levels of *Zmiz1* in murine retinal endothelial cells during postnatal development determined using the available bulk RNA sequencing data. **(E)** Representative images of P7 retinas stained with IB4 and Zmiz1 (A, artery; V, vein). Scale bars: 200 μm.

SUPPLEMENTARY FIGURE S2 Heterozygous loss of *Zmiz1* in ECs leads to defects in radial outgrowth and vascular density. **(A)** Representative images of whole-mount retinas stained with IB4 and α-SMA at P7. Scale bars: 1,000 μm. **(B,C)** Quantification of vascular outgrowth and vascular density in *Zmiz1* ^fl/wt^ (n = 8) and *Zmiz1* ^fl/wt;iECKO^ (n = 16) retinas at P7. Error bars represent mean ± s.e.m; two-tailed unpaired t-test. ns (not significant; P > 0.05), ***P < 0.001, ****P < 0.0001.

SUPPLEMENTARY FIGURE S3
*Zmiz1* ^f/f^ and *Zmiz1* ^fl/fl^; *Cdh5*-CreERT2 retinas without tamoxifen exhibit similar vascular parameters. **(A)** Representative images of whole-mount P7 retinas stained for IB4 (green) and alpha-SMOOTH MUSCLE ACTIN (α-SMA; red). Dotted white circle represents outgrowth in *Zmiz1* ^fl/fl^ retina. Scale bar, 1,000 μm. A, artery; V, vein. **(B-E)** Quantification of noted morphometric vascular parameters of *Zmiz1* ^fl/fl^ (n = 6) and *Zmiz1* ^iECKO^ (n = 5) retinas not subjected to tamoxifen. No significant differences were observed, except in vascular outgrowth. However, it is noted that the vascular outgrowth here in *Zmiz1* ^iECKO^ retinas is similar to *Zmiz1* ^f/f^ retinas with tamoxifen (Figure 2D). Error bars represent mean ± s.e.m; two-tailed unpaired t-test. ns (not significant; P > 0.05), *P = 0.0383.

SUPPLEMENTARY FIGURE S4 Endothelial *Zmiz1* is dispensable for vascular remodeling and maintenance during adulthood. **(A)** Schematic showing tamoxifen administration in 4 weeks old mice and analysis at P42 and P60. **(B)** Representative images of whole-mount retinas stained with IB4 at P42 and P60. Scale bars 1,000 μm. **(C)** Representative images of IB4+ vessels in superficial and deep layers of *Zmiz1* ^fl/fl^ and *Zmiz1* ^iECKO^ retinas at P42. Scale bars: 200 μm. **(D,E)** Quantification of superficial and deep vascular plexus density in *Zmiz1* ^fl/fl^ (n = 6) and *Zmiz1* ^iECKO^ (n = 8) retinas at P42. **(F,G)** Quantification of superficial and deep vascular plexus density in *Zmiz1* ^fl/fl^ (n = 7) and *Zmiz1* ^iECKO^ (n = 6) retinas at P60. Error bars represent mean ± s.e.m; two-tailed unpaired t-test. ns (not significant; P > 0.05).

SUPPLEMENTARY FIGURE S5 No changes in retinal vascular regression, pericyte coverage and EC proliferation and death are observed in *Zmiz1* mutant mice at P10. **(A)** Representative images of P10 retinas stained with Ki67, ERG, and IB4. Scale bars: 100 μm. **(B)** Quantification of Ki67^+^ proliferating ECs (ERG+) in *Zmiz1* ^fl/fl^ (n = 3) and *Zmiz1* ^iECKO^ (n = 7) mice at P10. **(C)** Representative images of P10 retinas stained with IB4 and CLEAVED CASPASE 3 (Cl-CASP3). Scale bars: 50 μm. **(D)** Quantification of Cl-CASP3^+^ apoptotic ECs in *Zmiz1* ^fl/fl^ (n = 3) and *Zmiz1* ^iECKO^ (n = 7) mice at P10. **(E)** Representative images of P10 retinas stained with IB4 and COLLAGEN IV (COL IV; white arrowheads indicate empty COL IV sleeves devoid of ECs). Scale bars 50 μm. **(F)** Quantification of empty COL IV sleeves in *Zmiz1* ^fl/fl^ (n = 3) and *Zmiz1* ^iECKO^ (n = 7) retinas at P10. **(G)** Representative images of P10 retinas stained with IB4 and NEURAL/GLIAL ANTIGEN 2 (NG2). Scale bars 100 μm. **(H)** Quantification of pericyte coverage in *Zmiz1* ^fl/fl^ (n = 3) and *Zmiz1* ^iECKO^ (n = 7) retinas at P10. Error bars represent mean ± s.e.m; two-tailed unpaired t-test. ns (not significant; P > 0.05).

SUPPLEMENTARY FIGURE S6 Whole-mount retinas from Figure 4. **(A)** Whole-mount *Zmiz1* ^fl/fl^ and *Zmiz1* ^iECKO^ P7 retinas from Figure 4A. IB4 (green) at P7.

SUPPLEMENTARY FIGURE S7 Expression of *Zmiz1* is unchanged during the neovascularization phase in the OIR model. **(A)** Schematic summary of the OIR model with RNA-seq analysis time points noted by black and blue arrow heads. Black arrows indicate time points from prior public data sets and blue arrows indicate time points from the current studies. **(B,C)** Quantifications of *Zmiz1* normal read counts obtained from publicly available RNA-seq data comparing P14 **(B)** and P17 **(C)** C57BL/6J retinas subjected to non-OIR (nOIR) and OIR environments (Pauleikhoff et al., 2023). Note no changes in *Zmiz1* expression levels during the neovascularization phase. nOIR_P14 (n = 7), OIR_P14 (n = 6), nOIR_P17 (n = 6) and OIR_P17 (n = 8) retinas. **(D,E)** Relative *Zmiz1* expression levels from non-OIR (nOIR) and OIR subjected C57BL/6J retinas at P12 **(D)** and P17 **(E)**, as determined by qRT-PCR and normalized to *β-actin*. nOIR_P12 (n = 4), OIR_P12 (n = 5), nOIR_P17 (n = 4) and OIR_P17 (n = 5) retinas. Error bars represent mean ± s.e.m; two-tailed unpaired t-test. ns (not significant).

SUPPLEMENTARY FIGURE S8 Loss of endothelial *Zmiz1* has no additional impact on the vasculature during the vaso-obliteration stage in the OIR model. **(A)** Schematic summary of the strategy implemented to assess *Zmiz1* contributions during the vaso-obliteration phase in the OIR model. **(B)** Representative whole-mount images of the vasculature immunolabeled with IB4 in *Zmiz1* ^fl/fl^-OIR (n = 8) and *Zmiz1* ^iECKO^-OIR (n = 9) P12 retinas. Yellow space demarcates the avascular area, while red markings indicate neovascular tufts within each retina (note the absence of tufts since this is prior to the neovascularization stage). Scale bars: 1,000 μm. **(C,D)** Quantification of the areas of vaso-obliteration (avascular space) and retinal neovascularization in *Zmiz1* ^fl/fl^-OIR (n = 8) and *Zmiz1* ^iECKO^-OIR (n = 8) P12 mice. Error bars represent mean ± s.e.m; two-tailed unpaired t-test. ns, not significant (P > 0.05).
