# Supplementary material for: Endothelial Zmiz1 modulates developmental angiogenesis in the retina
Source: Front Cell Dev Biol. 2025 Oct 1;13:1570587. doi: 10.3389/fcell.2025.1570587 (PMC12521193; doi:10.3389/fcell.2025.1570587)
Supplement: Supplementary file 1 [file DataSheet1.pdf]

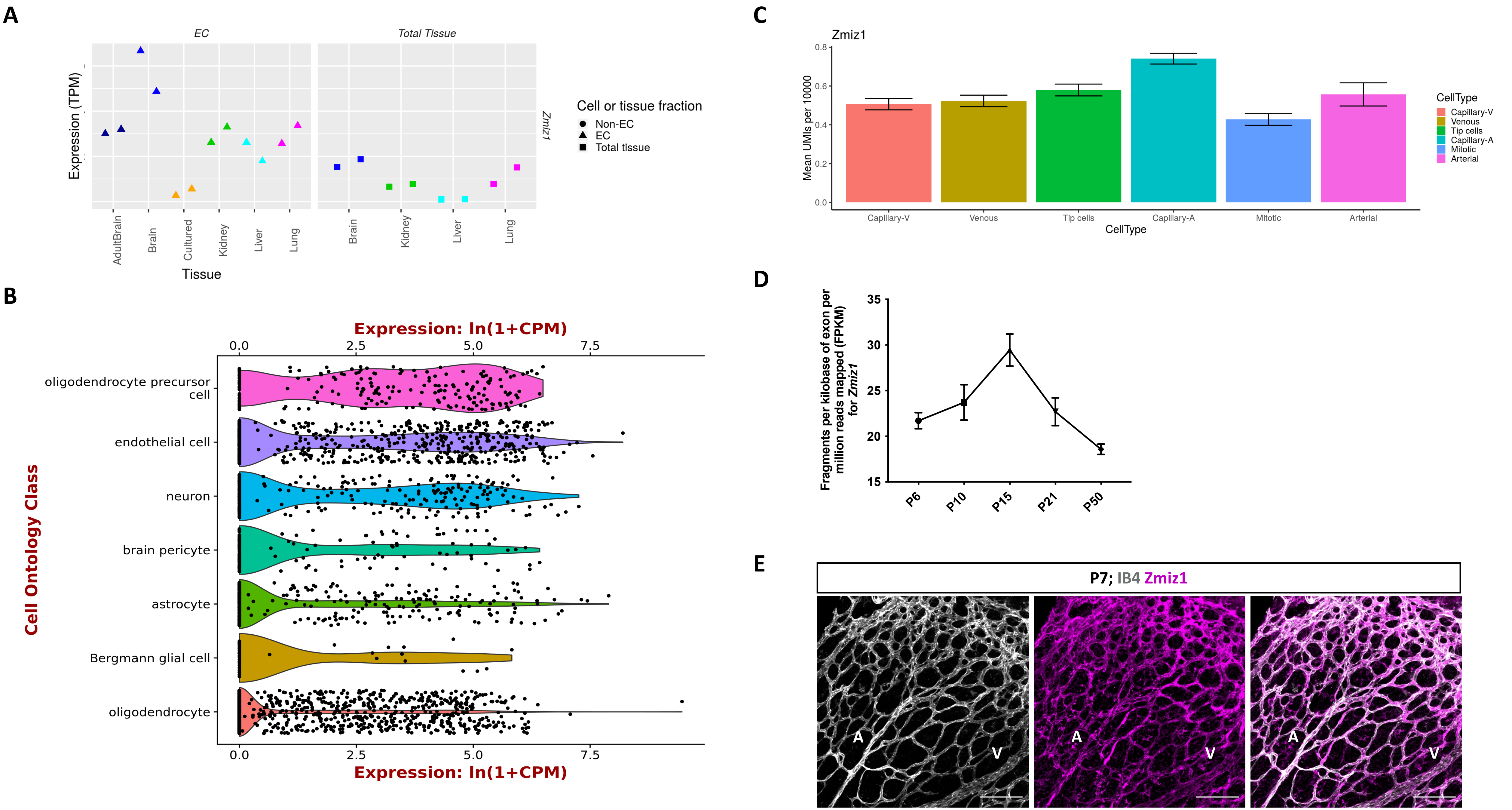
A
EC
Total Tissue
Expression (TPM)
Zmiz1
AdultBrain
Brain
Cultured
Kidney
Liver
Lung
Brain
Kidney
Liver
Lung
Tissue
Cell or tissue fraction
Non-EC
EC
Total tissue
B
Expression: ln(1+CPM)
0.0
2.5
5.0
7.5
Cell Ontology Class
oligodendrocyte precursor cell
endothelial cell
neuron
brain pericyte
astrocyte
Bergmann glial cell
oligodendrocyte
C
Zmiz1
Mean UMIs per 10000
0.0
0.2
0.4
0.6
0.8
Capillary-V
Venous
Tip cells
Capillary-A
Mitotic
Arterial
CellType
D
Fragments per kilobase of exon per million reads mapped (FPKM) for Zmiz1
15
20
25
30
35
P6
P10
P15
P21
P50
E
P7; IB4 Zmiz1
A
V

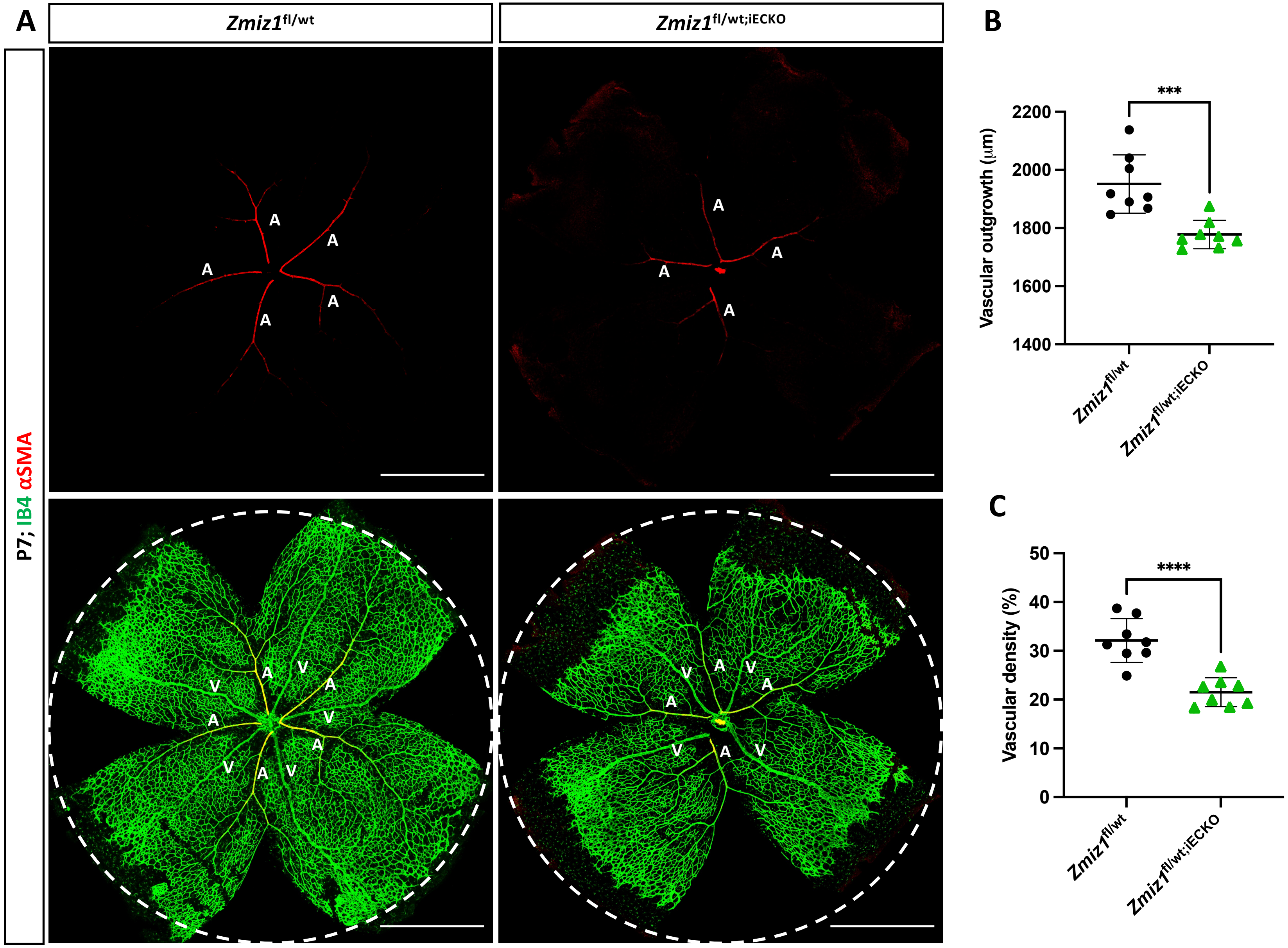

A
Zmiz1fl/wt
Zmiz1fl/wt;iECKO
P7; IB4 αSMA
A
V
B
***
Vascular outgrowth (μm)
2200
2000
1800
1600
1400
Zmiz1fl/wt
Zmiz1fl/wt;iECKO
C
****
Vascular density (%)
50
40
30
20
10
0
Zmiz1fl/wt
Zmiz1fl/wt;iECKO

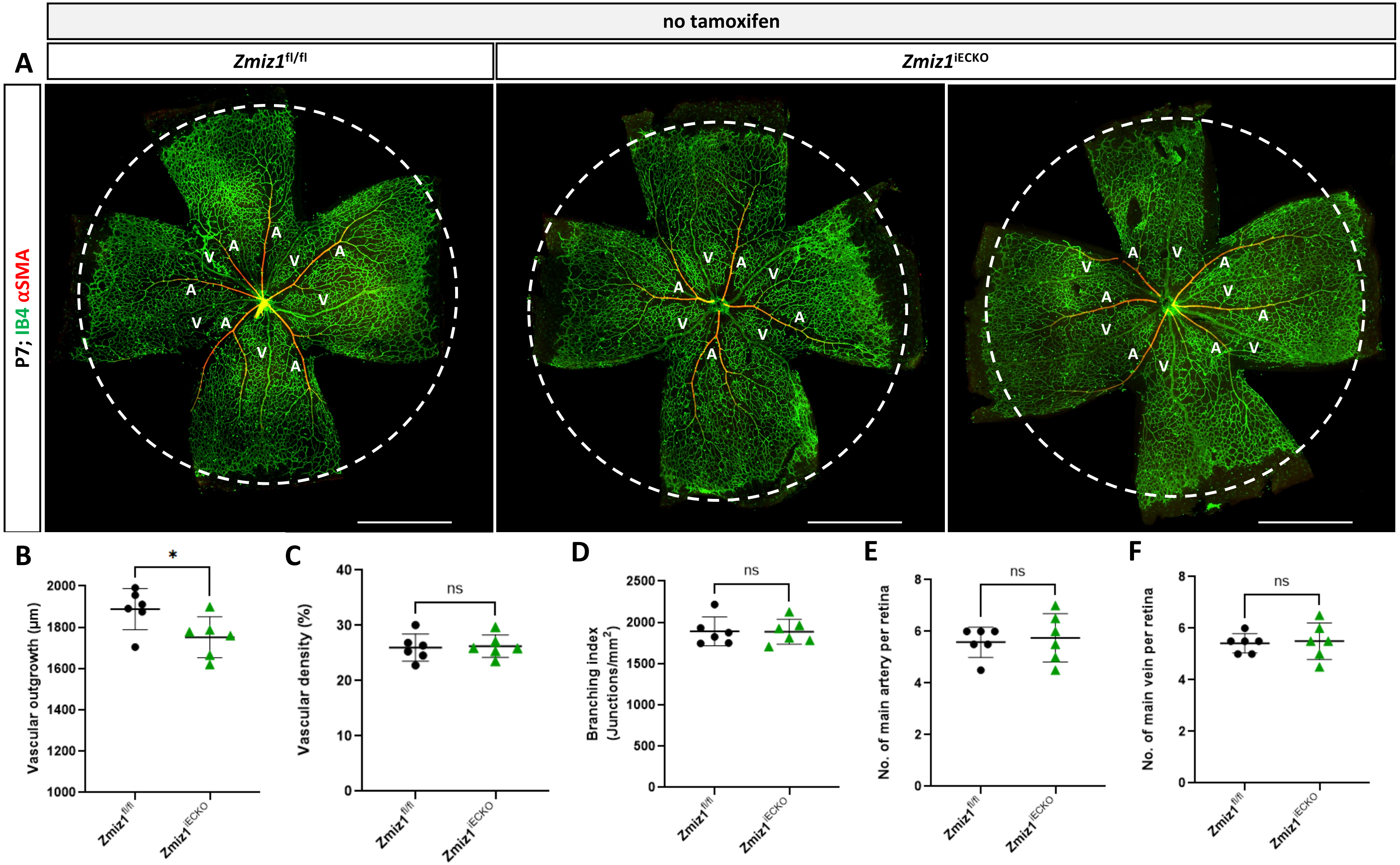

no tamoxifen
A
Zmiz1fl/fl
Zmiz1iECKO
P7; IB4 αSMA
A
V
B
*
Vascular outgrowth (µm)
2000
1800
1600
1400
1200
1000
Zmiz1fl/fl
Zmiz1iECKO
C
ns
Vascular density (%)
40
30
20
10
0
D
ns
Branching index (Junctions/mm2)
2500
2000
1500
1000
500
0
E
ns
No. of main artery per retina
8
6
4
2
0
F
ns
No. of main vein per retina

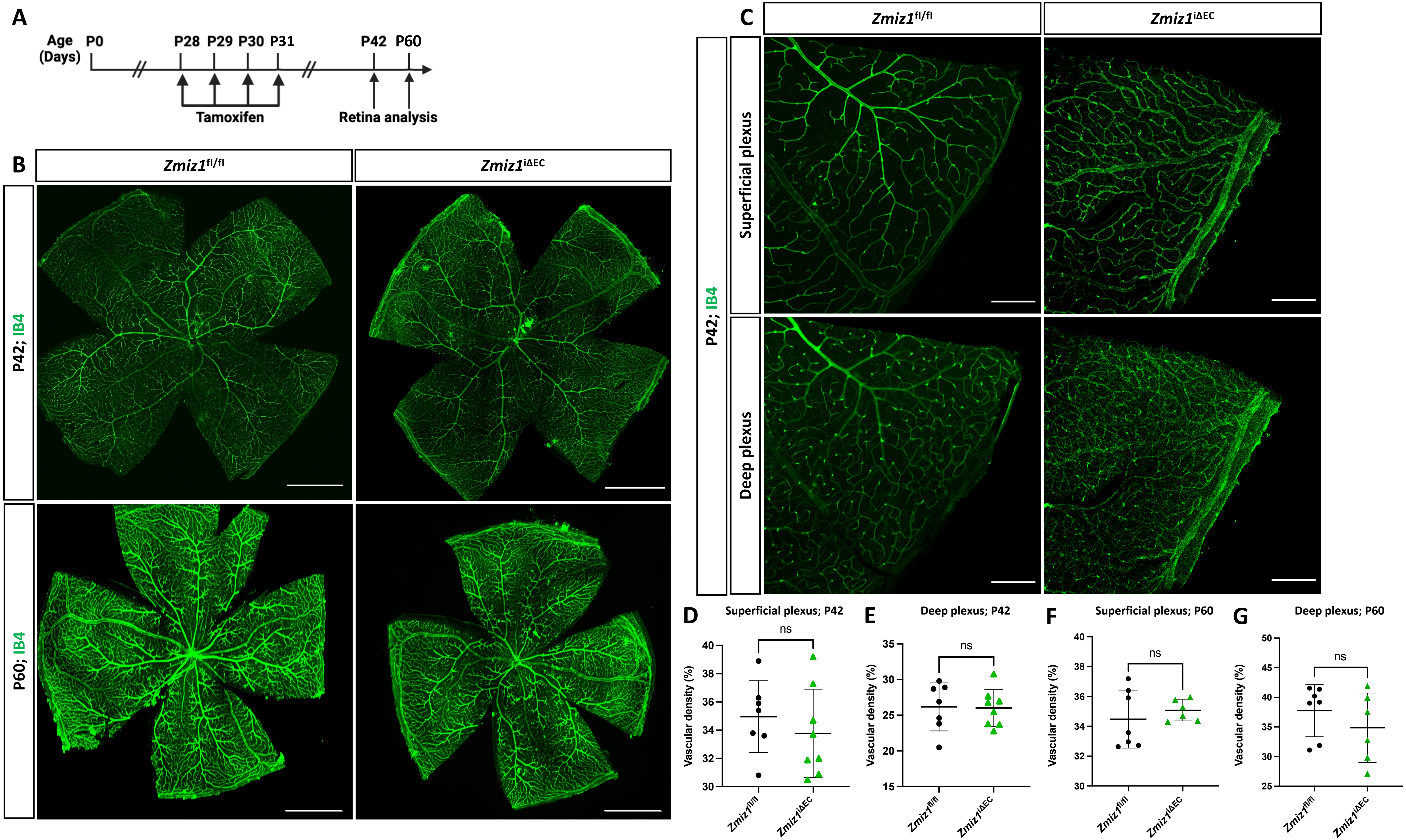

A
Age (Days)
P0
P28
P29
P30
P31
P42
P60
Tamoxifen
Retina analysis
B
Zmiz1fl/fl
Zmiz1iΔEC
P42; IB4
P60; IB4
C
Zmiz1fl/fl
Zmiz1iΔEC
P42; IB4
Superficial plexus
Deep plexus
D
Superficial plexus; P42
E
Deep plexus; P42
F
Superficial plexus; P60
G
Deep plexus; P60
ns
Vascular density (%)
Zmiz1fl/fl
Zmiz1iΔEC

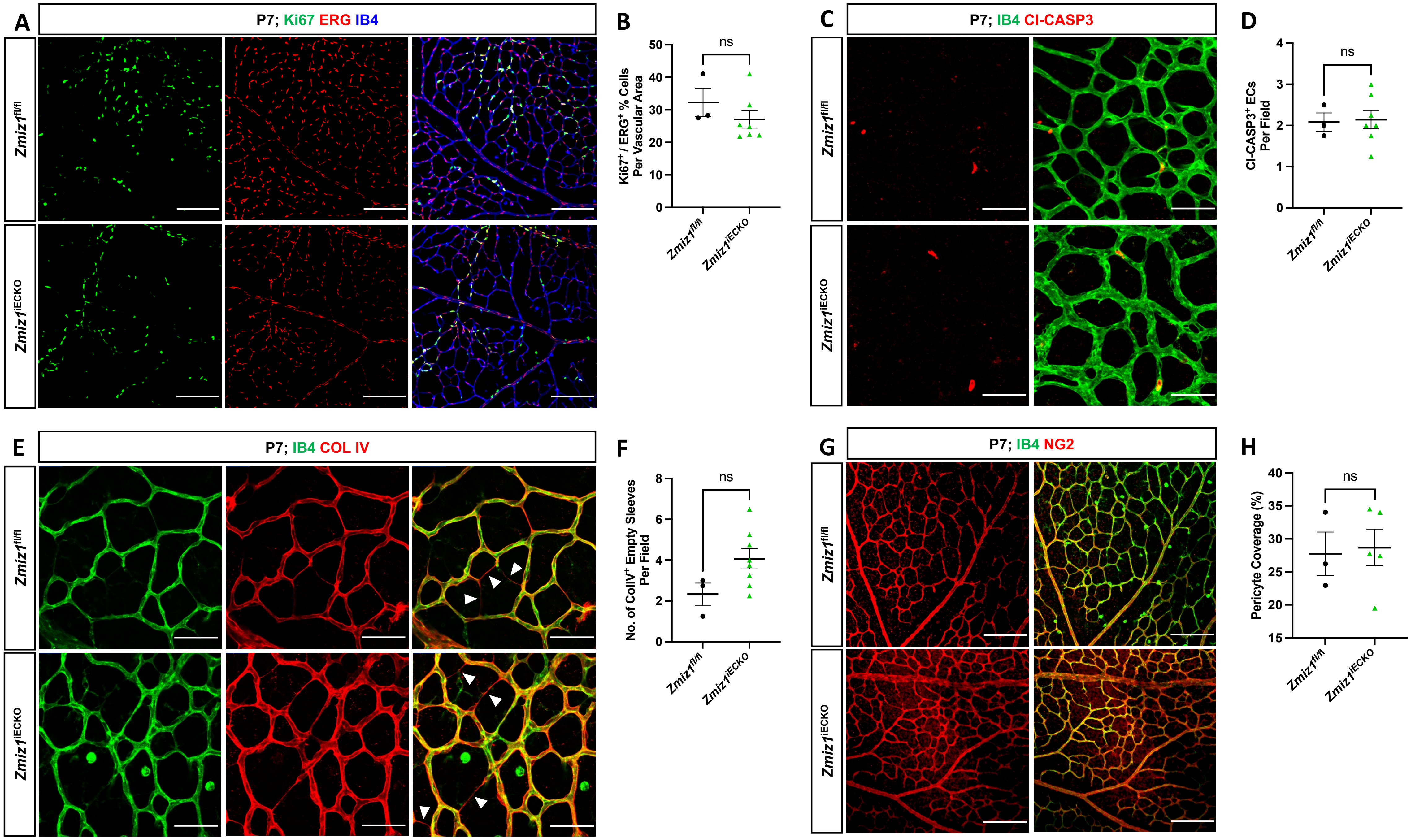

A
P7; Ki67 ERG IB4
Zmiz1fl/fl
Zmiz1iECKO
B
ns
Ki67+ / ERG+ % Cells Per Vascular Area
50
40
30
20
10
0
Zmiz1fl/fl
Zmiz1iECKO
C
P7; IB4 Cl-CASP3
D
ns
Cl-CASP3+ ECs Per Field
4
3
2
1
0
E
P7; IB4 COL IV
F
ns
No. of ColIV+ Empty Sleeves Per Field
8
6
4
2
0
G
P7; IB4 NG2
H
ns
Pericyte Coverage (%)
40
35
30
25
20
15

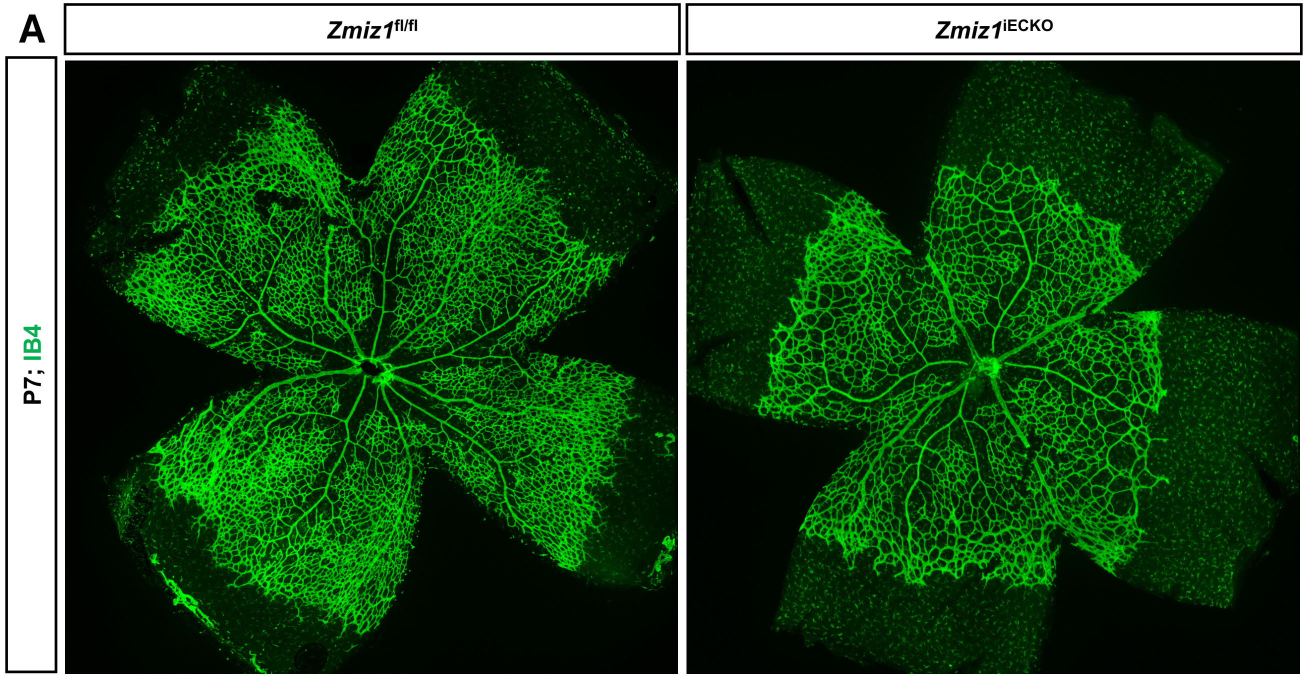

A
Zmiz1fl/fl
Zmiz1iECKO
P7; IB4

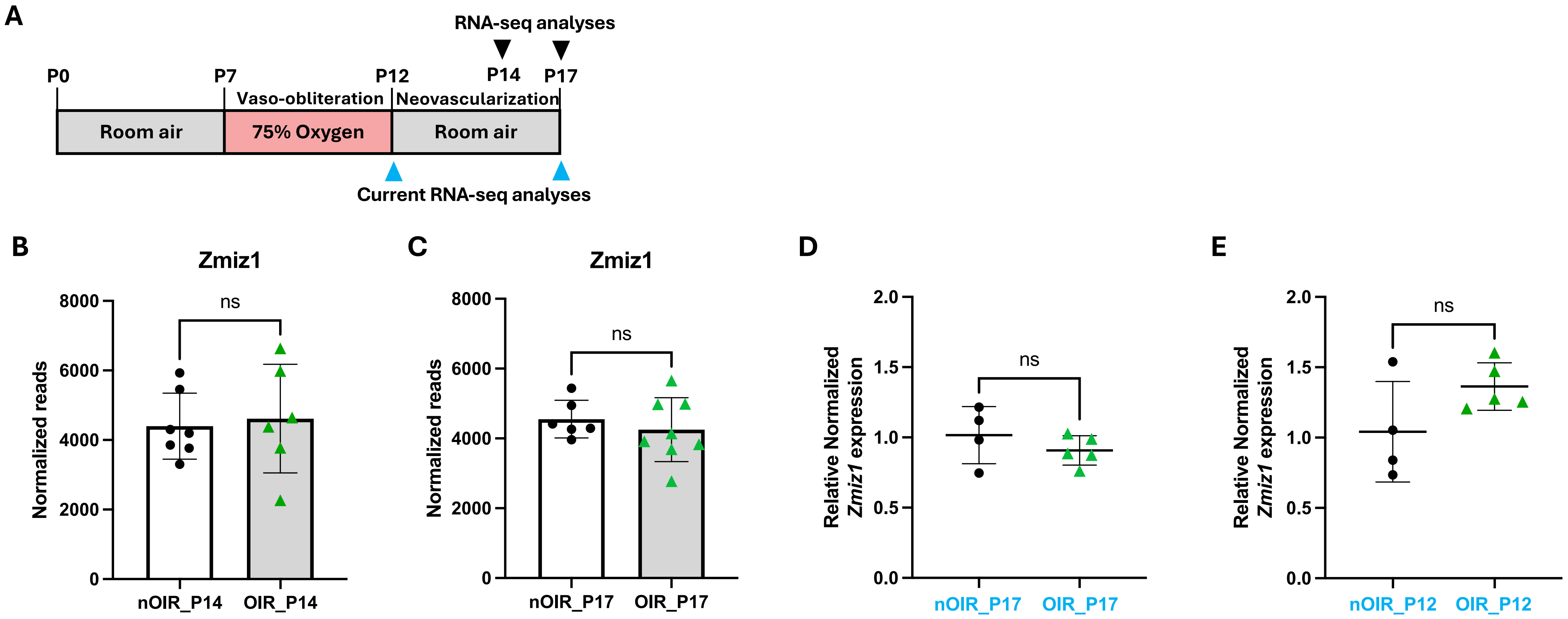

A
RNA-seq analyses
P0
P7
P12
P14
P17
Vaso-obliteration
Neovascularization
Room air
75% Oxygen
Room air
Current RNA-seq analyses
B
Zmiz1
ns
Normalized reads
8000
6000
4000
2000
0
nOIR_P14
OIR_P14
C
Zmiz1
ns
Normalized reads
8000
6000
4000
2000
0
nOIR_P17
OIR_P17
D
ns
Relative Normalized
Zmiz1 expression
2.0
1.5
1.0
0.5
0.0
nOIR_P17
OIR_P17
E
ns
Relative Normalized
Zmiz1 expression
2.0
1.5
1.0
0.5
0.0
nOIR_P12
OIR_P12

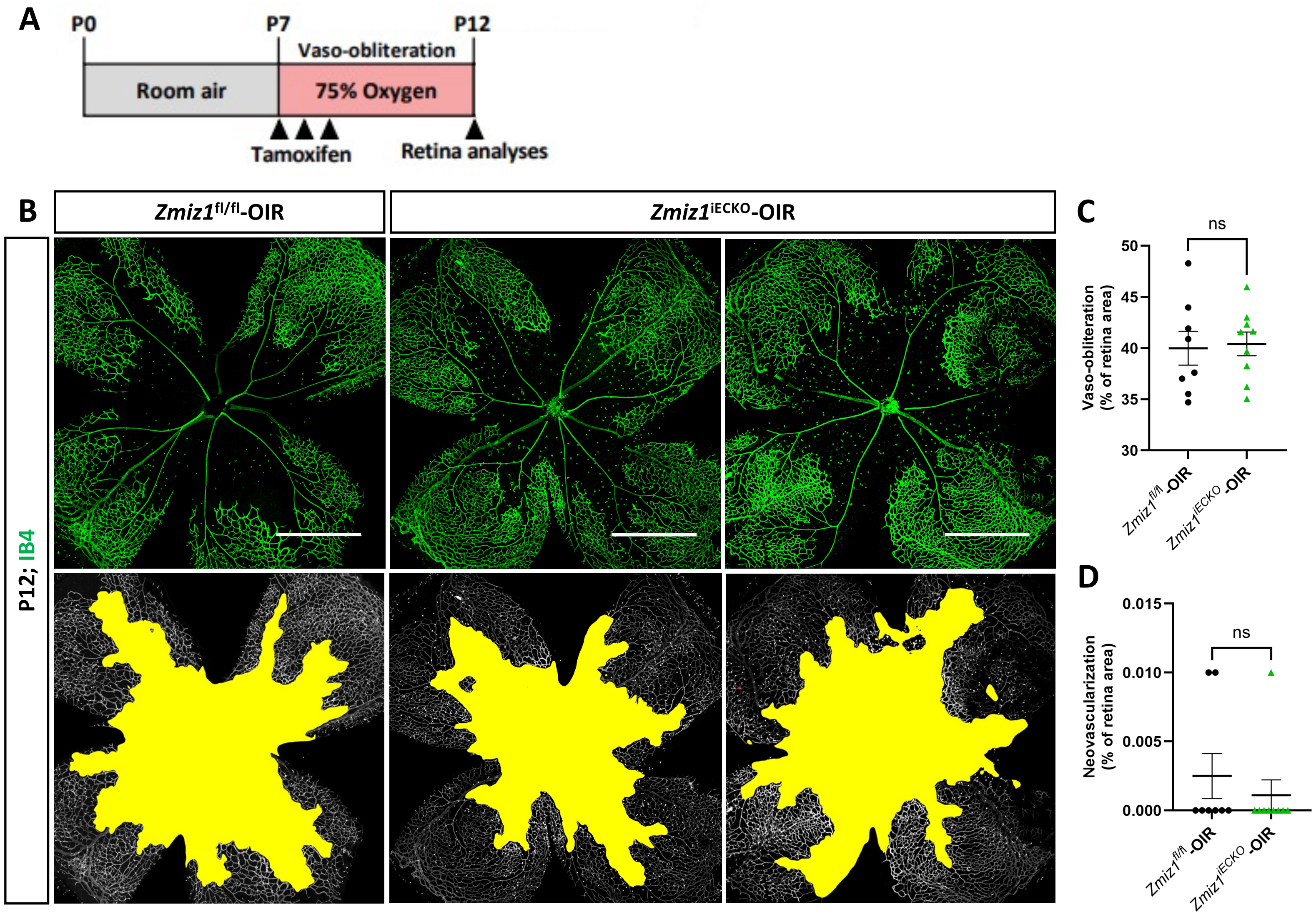
A
P0
P7
P12
Vaso-obliteration
Room air
75% Oxygen
Tamoxifen
Retina analyses
B
Zmiz1fl/fl-OIR
Zmiz1iECKO-OIR
P12; IB4
C
ns
Vaso-obliteration (% of retina area)
50
45
40
35
30
Zmiz1fl/fl-OIR
Zmiz1iECKO-OIR
D
ns
Neovascularization (% of retina area)
0.015
0.010
0.005
0.000
Zmiz1fl/fl-OIR
Zmiz1iECKO-OIR
